# Supplementary material for: Predicting chromatin conformation contact maps
Source: PLoS One. 2025 Sep 29;20(9):e0331124. doi: 10.1371/journal.pone.0331124 (PMC12478923; doi:10.1371/journal.pone.0331124)
Supplement: S1 File — (PDF) [file pone.0331124.s001.pdf]

Supplement to "Predicting chromatin conformation contact maps"

Alan Min[1], Jacob Schreiber[2], Anshul Kundaje[2], and William S. Noble[3,4]

[1]Department of Statistics, University of Washington
[2]Department of Genetics, Stanford University
[3]Department of Genome Sciences, University of Washington
[4]Paul G. Allen School of Computer Science and Engineering, University of Washington

S1 Sphinx at 10kb resolution

We trained the Sphinx model at 10kb resolution on chromosome 19. We used the same model architecture, normalization, and pruning method as in the standard Sphinx model. We conducted a hyperparameter search similar to that described in Section 2.1, but only 10 random hyperparameter sets were tested because the 10kb model was much more computationally expensive. Whereas at 100kb the matrices were 556×556 bins, at 10kb each matrix was 5278×5278 bins. We trained the 10kb model for 50 epochs, ultimately choosing the best validation epoch.

In this experiment, each of the combinations performed similarly to the cross-mean baseline (Figure S1A). The best performing model used 256 celltype factors, 64 assay factors, 256 position factors, 256 distance factors, 16 hidden nodes, 8 hidden layers, 0.6 dropout, and a learning rate of 0.0005. We further compared the test set performance of Sphinx at 10kb versus the baseline mean model and found that Sphinx performed similarly to the baseline model (Figure S1B). We hypothesize that the reason Sphinx is not able to outperform the mean model is the high sparsity of the data at 10kb resolution compared to at 100kb resolution, where the matrices were dense in all of our observed data (Figure S1C).

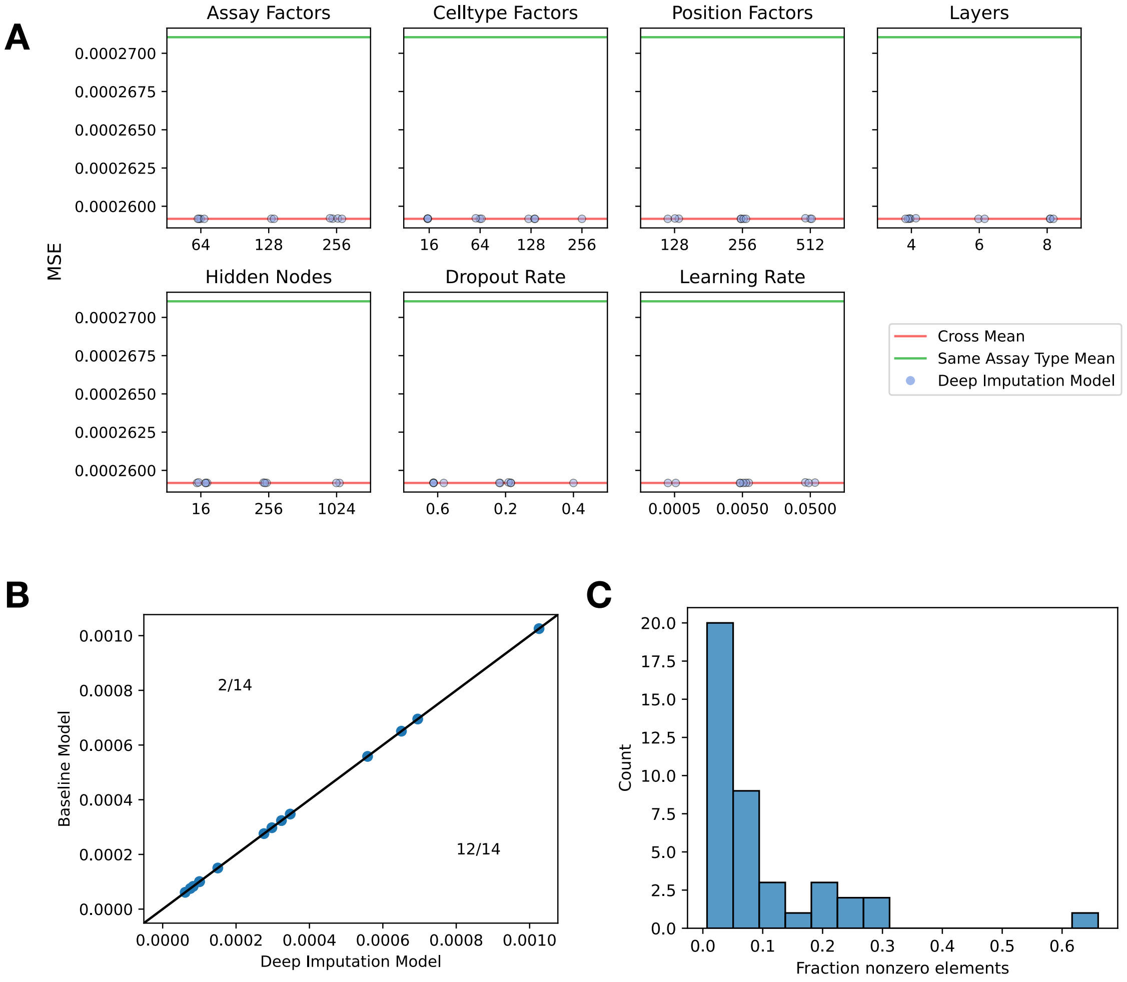

Figure S1: **Sphinx does not offer significant improvement at 10kb resolution** (a) Sphinx was trained using 10 randomly selected settings of seven different hyperparameters. Each panel plots the validation set MSE (x-axis) for various values of a particular hyperparameter (y-axis). MSEs are shown as horizontal lines for the baseline methods: cross-mean (red line) and same assay type (green). Training is done at 10kb resolution on chromosome 19. (b) Test-set MSE is shown comparing the deep imputation model to the cross-mean baseline. The number of points on each side of the $y = x$ line is annotated. (c) A histogram of the fraction on nonzero elements at 10kb resolution for every available dataset is shown.

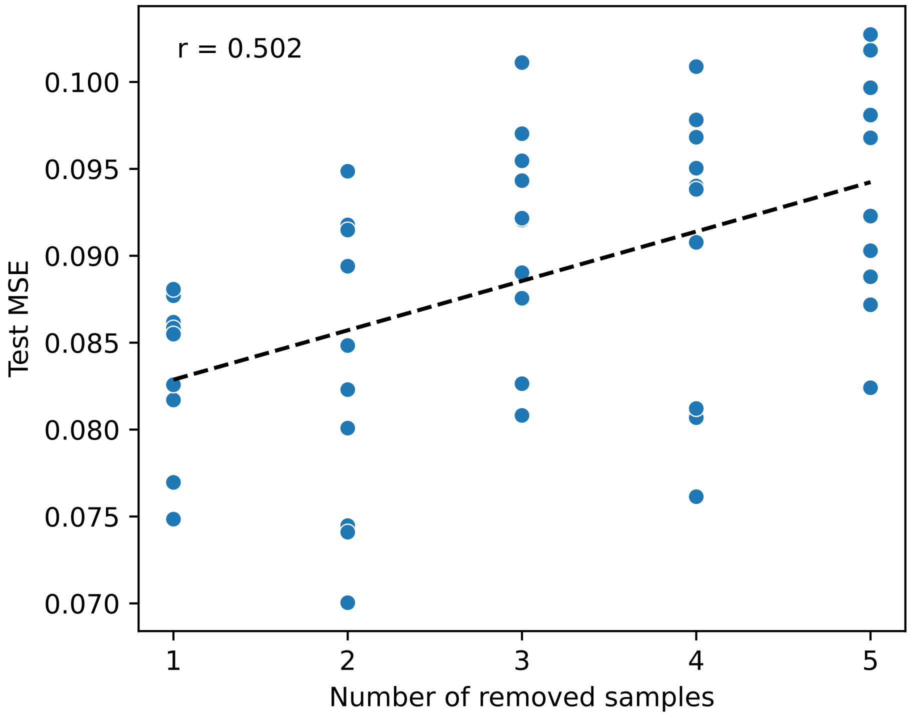

Figure S2: **MSE increases as the number of training examples decreases**. One to five samples were randomly removed (X-axis) from the training data so that there was always at least one training example of each celltype and assay were still present in the training set, and the and test MSE was recorded. This was done for 10 random starting seeds.

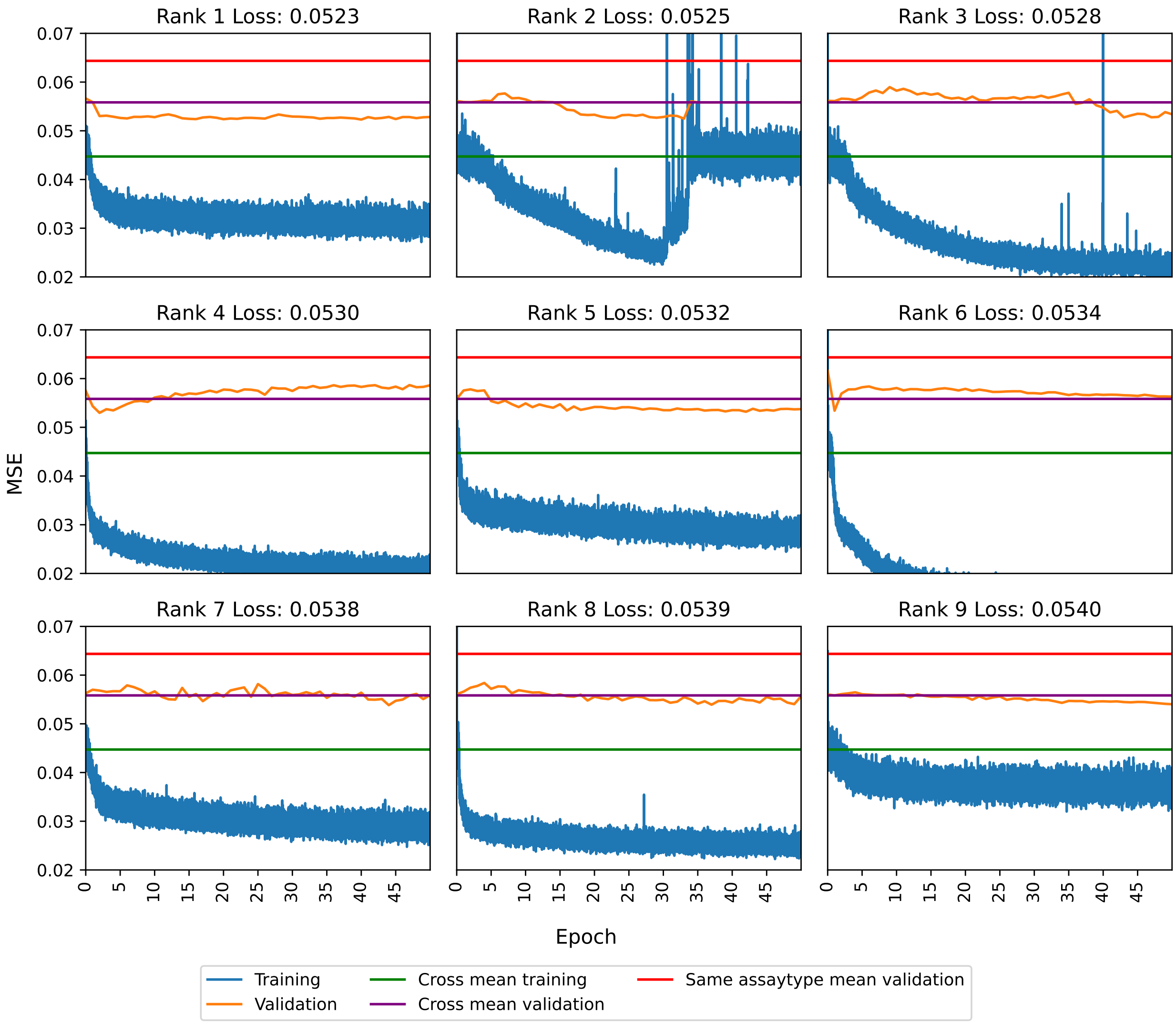

Figure S3: **Loss curves demonstrate sufficient training epochs.** The loss curves for the 9 lowest hyperparameter combinations are shown. The associated hyperparameter combinations are shown in Table S1. The Sphinx training loss (blue), Sphinx validation loss (orange), cross-mean baseline training loss (green), cross-mean validation loss (purple), and same-assay validation loss (red) are shown.

Rank	Loss	Assay	Cell type	Position	Layers	Nodes	Dropout	Learning Rate
1	0.0523	128	16	128	4	256	0.4	0.0005
2	0.0524	64	64	512	6	1024	0.2	0.0050
3	0.0527	64	128	128	4	1024	0.2	0.0050
4	0.0529	128	16	1024	4	256	0.2	0.0005
5	0.0531	256	256	128	6	1024	0.4	0.0005
6	0.0534	16	256	1024	2	1024	0.2	0.0005
7	0.0538	128	16	128	8	1024	0.4	0.0005
8	0.0539	256	128	128	4	256	0.2	0.0050
9	0.0540	128	64	256	4	256	0.6	0.0005

Table S1: **Results of the hyperparameter search.** The table lists the best-performing nine hyperparameter settings, with the corresponding loss values.

Biosource	Assay Type	Accession	Generating Lab
192627	Dilution Hi-C	4DNESYPKLMAM	Erez Lieberman Aiden
192627	in situ Hi-C	4DNESECNR4O8	Erez Lieberman Aiden
CC-2551	Dilution Hi-C	4DNESUB35TII	Erez Lieberman Aiden
CC-2551	in situ Hi-C	4DNESIE5R9HS	Erez Lieberman Aiden
GM12878	Dilution Hi-C	4DNESLLTENG9	Bing Ren
GM12878	DNA SPRITE	4DNESI1U7ZW9	Mitchell Guttman
GM12878	in situ ChIA-PET CTCF protein	4DNES7IB5LY9	Yijun Ruan
GM12878	in situ ChIA-PET RNA Pol II	4DNESZ25MOZV	Yijun Ruan
GM12878	in situ Hi-C	4DNESPXW8XHY	Erez Lieberman Aiden
GM12878	PLAC-seq H3K4me3	4DNESL3LFLGI	Bing Ren
H1-hESC	in situ ChIA-PET CTCF protein	4DNESR9S8R38	Yijun Ruan
H1-hESC	in situ ChIA-PET RNA Pol II	4DNESNYUGLUN	Yijun Ruan
H1-hESC	in situ Hi-C	4DNES2M5JIGV	Job Dekker
H1-hESC	Micro-C	4DNES21D8SP8	Job Dekker
H1-hESC	PLAC-seq H3K4me3	4DNESQMO66LZ	Bing Ren
HeLa cell line	Dilution Hi-C	4DNESWMJBQMR	Jan-Michael Peters
HeLa cell line	DNase Hi-C	4DNESGEEV6TJ	Todd Waldman
HeLa cell line	in situ Hi-C	4DNESEDV9YMX	Job Dekker
HeLa cell line	Micro-C	4DNESA5PN8AB	Job Dekker
HFF-hTERT	Dilution Hi-C	4DNES9L4AK6Q	Job Dekker
HFF-hTERT	in situ Hi-C	4DNESB6MNCFE	Job Dekker
HFF-hTERT	Micro-C	4DNESEGKQY7I	Job Dekker
HFFc6 (Tier 1)	DNA SPRITE	4DNESJYGTI8S	Mitchell Guttman
HFFc6 (Tier 1)	in situ ChIA-PET CTCF protein	4DNESCQ7ZD21	Yijun Ruan
HFFc6 (Tier 1)	in situ ChIA-PET RNA Pol II	4DNESI1WZ5HT	Yijun Ruan
HFFc6 (Tier 1)	in situ Hi-C	4DNES2R6PUEK	Job Dekker
HFFc6 (Tier 1)	Micro-C	4DNESWST3UBH	Job Dekker
HFFc6 (Tier 1)	PLAC-seq H3K4me3	4DNESIF5UIQE	Bing Ren
HUVEC cell	Dilution Hi-C	4DNESOSE2FYZ	Erez Lieberman Aiden
HUVEC cell	in situ Hi-C	4DNESEW5JLUC	Erez Lieberman Aiden
IMR-90	Dilution Hi-C	4DNESSM1H92K	Erez Lieberman Aiden
IMR-90	in situ Hi-C	4DNES1ZEJNRU	Erez Lieberman Aiden
WTC-11	in situ ChIA-PET CTCF protein	4DNES8MZ76GP	Yijun Ruan
WTC-11	in situ ChIA-PET RNA Pol II	4DNESRRTL4BU	Yijun Ruan
WTC-11	in situ Hi-C	4DNESPDEZNWX	Job Dekker
WTC-11	Micro-C	4DNESODGV2V2	Job Dekker
WTC-11	PLAC-seq H3K4me3	4DNESDRL4ZKM	Bing Ren
WTC-11 AAVS1-GFP C28	DNase Hi-C	4DNES8BLXVP5	Chuck Murry
WTC-11 AAVS1-GFP C28	in situ Hi-C	4DNESJ7S5NDJ	Job Dekker
WTC-11 AAVS1-GFP C28	Micro-C	4DNESAGG7EUC	Job Dekker
WTC-11 AAVS1-GFP C28	PLAC-seq H3K4me3	4DNESIZ5TTHO	Bing Ren

Table S2: **4D Nucleome data sets used in this study.**

S2 Imputing Loops

Another important aspect of HiC data is predicting loops, which are regions of the genome that are brought together in three dimensional space through loop extrusion. These loops have biological significance in modulating gene expression, and many methods have been proposed to detect loops [1–3]. It is hence of interest to determine whether loops can be imputed through Sphinx. We used the HiCDetectLoops package [1] to detect loops from observed, mean model, and Sphinx predictions at 10kb resolution. We transformed our contact matrices back into raw counts by transforming $\exp(y) - 1$ for both the observed and predicted matrices. We used the following command, which uses the recommended settings from the HiCDetectLoops documentation, including the following parameters: `--maxLoopDistance 2000000 --windowSize 10 --peakWidth 6 --pValuePreselection 0.05 --pValue 0.05`. We detected loops in the observed and predicted matrices for chromosome 19 for each of the 14 entries in our test set.

When using the HiCDetectLoops recommended settings on the WTC-11/ChIA-PET RNA Pol II data, we detected 2 loops in the observed matrices, 9 loops in the Sphinx imputations, and 7 in the mean model. We considered loops to be the same if the start and end of the loops were within 50kb of each other. None of the loops were shared between the observed and either the mean or the Sphinx predictions (Table S3); however, one loop was detected within 1 Mb in both the observed and the imputed matrices at 40Mb. All loops detected in the mean model were also detected in the Sphinx prediction. We also detected 18 loops in the observed WTC-11/ChIA-PET CTCF experiments and 4 loops in the HeLa/DNAse experiments, but no

			Sphinx		Mean model	
Assay	Biosample	Observed loops	Predicted loops	Intersection	Predicted loops	Intersection
in situ ChIA-PET RNA Pol II	WTC 11	2	9	0	7	0
in situ ChIA-PET CTCF	WTC 11	18	0	0	0	0
HeLA	DNAse	4	0	0	0	0
DNA SPRITE	GM12878	0	13	0	13	0

Table S3: **Mean model and Sphinx loop predictions do not agree with the observed loops predictions.** We report all test-set experiments that showed any loop calls, as well as the number of loops that were in the intersection between Sphinx and observed or the number of loops in the intersection of the mean model and observed data, where loops were considered the same if their starts and ends were both within 50kb of each other. All other samples had no detected loops.

loops were detected in the imputed matrices. We detected 13 loops in the imputed mean model and Sphinx GM12878/DNA SPRITE matrices, but no loops were detected in the observed data. The same contact matrices had loops detected using either the recommended or relaxed HiCDetectLoops commands.

We also used relaxed settings in HiCDetectLoops to attempt to find additional loops, changing maxLoopDistances from 2,000,000 to 5,000,000, and the p-value parameters from 0.05 to 0.1. However, using this approach we still found no matching loops between the observed and imputed matrices using either Sphinx or the mean model. Because loops are a high resolution feature, we expect that further refinement may produce more concordant loop calls.

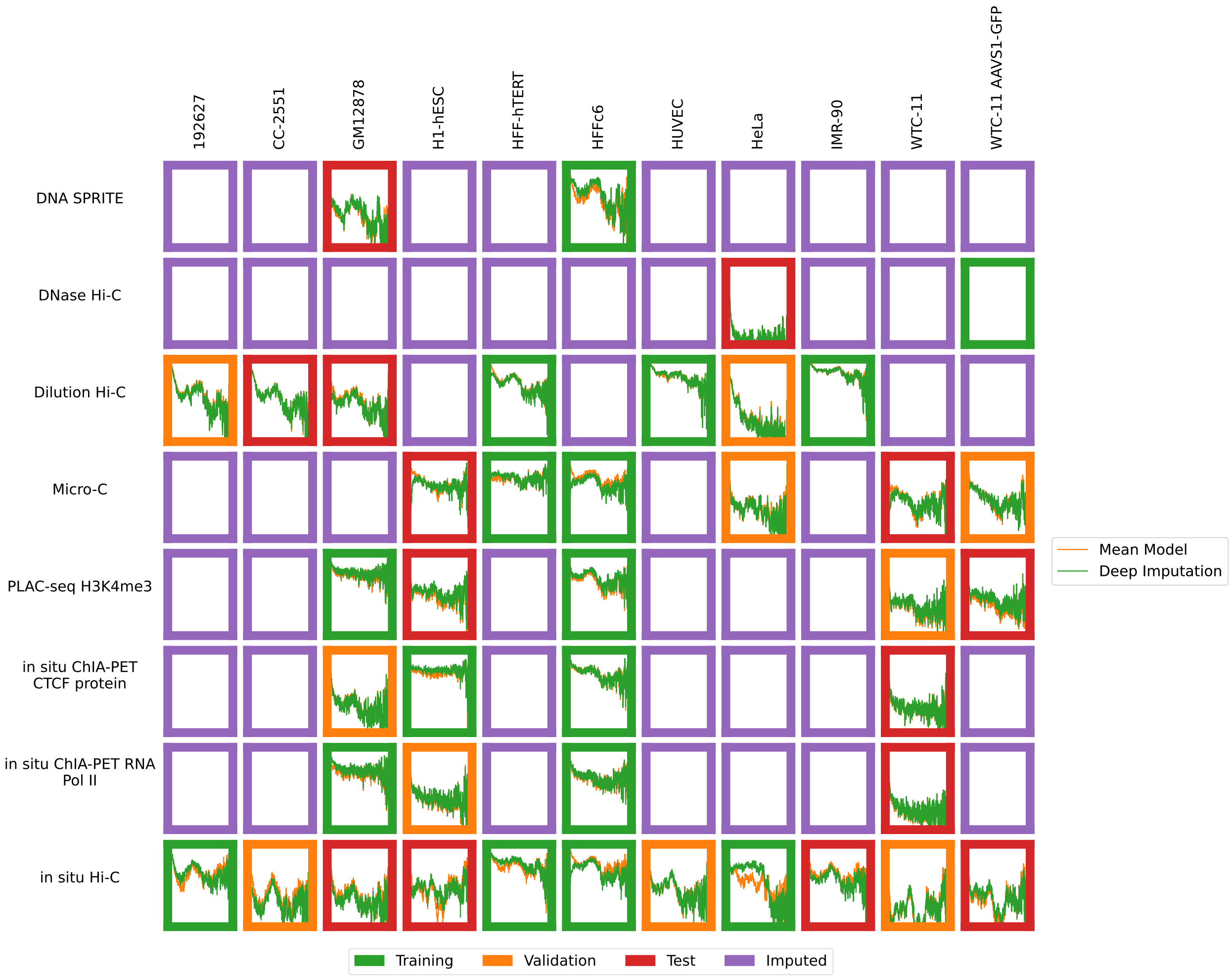

Figure S4: **Pearson correlation by distance.** Pearson correlation (y-axis, ranging from 0-1) is plotted against the genomic distance of the two positions (x-axis) [4]. The correlation is between either the mean model (orange lines) or Sphinx (green lines) compared to the observed contact matrices. The border of each panel indicates the data split that the matrix was assigned to.

References

[1] Joachim Wolff, Rolf Backofen, and Björn Grüning. Loop detection using hi-c data with hicexplorer. *Gigascience*, 11:giac061, 2022.

[2] Abbas Roayaei Ardakany, Halil Tuvan Gezer, Stefano Lonardi, and Ferhat Ay. Mustache: multi-scale detection of chromatin loops from Hi-C and micro-C maps using scale-space representation. *Genome Biology*, 21:1–17, 2020.

[3] N. C. Durand, M. S. Shamim, I. Machol, S. S. Rao, M. H. Huntley, E. S. Lander, and E. L. Aiden. Juicer provides a one-click system for analyzing loop-resolution Hi-C experiments. *Cell Systems*, 3(1):95–98, 2016.

[4] Vinícius G Contessoto, Ryan R Cheng, Arya Hajitaheri, Esteban Dodero-Rojas, Matheus F Mello, Erez Lieberman-Aiden, Peter G Wolynes, Michele Di Pierro, and José N Onuchic. The nucleome data bank: web-based resources to simulate and analyze the three-dimensional genome. *Nucleic Acids Research*, 49(D1):D172–D182, 2021.
