# Supplementary material for: Predicting chromatin conformation contact maps
Source: PLoS One. 2025 Sep 29;20(9):e0331124. doi: 10.1371/journal.pone.0331124 (PMC12478923; doi:10.1371/journal.pone.0331124)
Supplement: Attachment — Submitted filename: review_response.pdf [file pone.0331124.s002.pdf]

Review response for "Predicting chromatin conformation contact maps"

Alan Min[1], Jacob Schreiber[2], Anshul Kundaje[2], and William Stafford Noble[3,4]

[1]Department of Statistics, University of Washington
[2]Department of Genetics, Stanford University
[3]Department of Genome Sciences, University of Washington
[4]Paul G. Allen School of Computer Science and Engineering, University of Washington

We appreciate the constructive suggestions made by all three reviewers, and we apologize for the delay in submitting this revised manuscript. The first author, Alan Min, has graduated and hence has limited availability to work on this project. Please see below for our detailed responses to each of the critiques. Where applicable, we have included the revised text in blue.

Reviewer 1

The authors present a machine learning approach for a novel task which consists in imputing unseen contact maps given a set of available contact maps. This is very interesting in the context of large 3D genome mapping projects such as 4DN (or ENCODE) for which 3D genome is not experimentally mapped for all conditions due to experimental cost and complexity, and there is a need to impute the missing conditions. However, the major limit I can see from the proposed method is the very low resolution of the predictions (100kb), which makes the method not as useful it could be in practice. Also, many results are not enough discussed (the result section is very thin and it is quite unusual). I believe the work is great and deserve some efforts to improve the paper writing and the github!

Thank you for this positive assessment of the potential impact of our work. We have addressed your concerns as outlined below.

Major revisions

1. I believe the low resolution will reduce the utility of the approach. However, I do believe this task can be done at a higher resolution if the authors follows a simple "hierarchical" approach as follows (here the aim of my suggestion is not to ask the authors to completely change their method but instead minor changes to adapt to high resolution): in addition of the prediction at low resolution, the authors could predict at mid resolution (25kb) and high resolution (5kb) by setting a distance limit, thereby imputing band matrices.

We made some efficiency improvements in our code to use our model on the 10kb data and were able to make some progress. At this resolution, training the model is more computationally intensive. As before, we trained our model using 50 epochs and selected the best model using the validation set. Because training now takes more time and memory, we were only able to try 10 hyperparameter combinations for the 10kb model, but we were able to achieve similar performance to the baseline model. One potential reason that we don't see improvements over the baseline model is that the sparsity of the data at 10kb is much greater

than at 100kb. We updated some of the main text to reflect these additional results.

We primarily trained the model on one chromosome (chromosome 19 for most analyses, and chromosome 1 in Section 3.4) at 100kb resolution. We also experimented with training our model at 10kb resolution but were unable to improve upon the baseline method (Section S1).

We also added a supplemental section to address these results:

S1 Sphinx at 10kb resolution

We trained the Sphinx model at 10kb resolution on chromosome 19. We used the same model architecture, normalization, and pruning method as in the standard Sphinx model. We conducted a hyperparameter search similar to that described in Section **??**, but only 10 random hyperparameter sets were tested because the 10kb model was much more computationally expensive. Whereas at 100kb the matrices were 556×556 bins, at 10kb each matrix was 5278×5278 bins. We trained the 10kb model for 50 epochs, ultimately choosing the best validation epoch.

In this experiment, each of the combinations performed similarly to the cross-mean baseline (Figure S1A). The best performing model used 256 celltype factors, 64 assay factors, 256 position factors, 256 distance factors, 16 hidden nodes, 8 hidden layers, 0.6 dropout, and a learning rate of 0.0005. We further compared the test set performance of Sphinx at 10kb versus the baseline mean model and found that Sphinx performed similarly to the baseline model (Figure S1B). We hypothesize that the reason Sphinx is not able to outperform the mean model is the high sparsity of the data at 10kb resolution compared to at 100kb resolution, where the matrices were dense in all of our observed data (Figure S1C).

2. Hi-C data is intrinsically hierarchical with a high percentage of zeroes at large distance when the resolution is high, meaning that experiments themselves cannot map well contacts at large distances for high resolutions, so there is no need that the proposed method could do it. If sparse tensor factorization could be used it could help to predict the band inside the matrix with a reasonable computational cost. I am not a specialist in this field, but I could see such methods exist for band matrices.

We agree that a sparse matrix approach could potentially improve our method and make it scalable to finer resolutions. However, presently there is limited support for sparse matrices in PyTorch, which was the main tool we used to build Sphinx.

3. on the same idea, I do believe that combining imputation at mid resolution (25kb) with Hi-C data augmentation could also be used to predict at high resolution (5kb). Same idea for 100kb to augment data to get 25kb.

This is an interesting idea that could potentially help address our sparsity problems. However, implementing an imputation model and integrating it into the Sphinx code would be a significant change that we currently do not have the resources to pursue, since it would involve first developing the model, training and validating the imputation model, tuning the imputation model, and then including the results when retraining the Sphinx model.

4. I believe that results are not enough discussed and must be split into subsections. There are only 2 result subsections!! Usually, we expect at least 3-4. I would say that one more subsection is very needed and one more would be appreciated to discuss more the results. There are so many figures that fits to subsection 3.2.

We have added an additional subsection to discuss the performance of Sphinx on chromosome 1 and at 10kb resolution in chromosome 19.

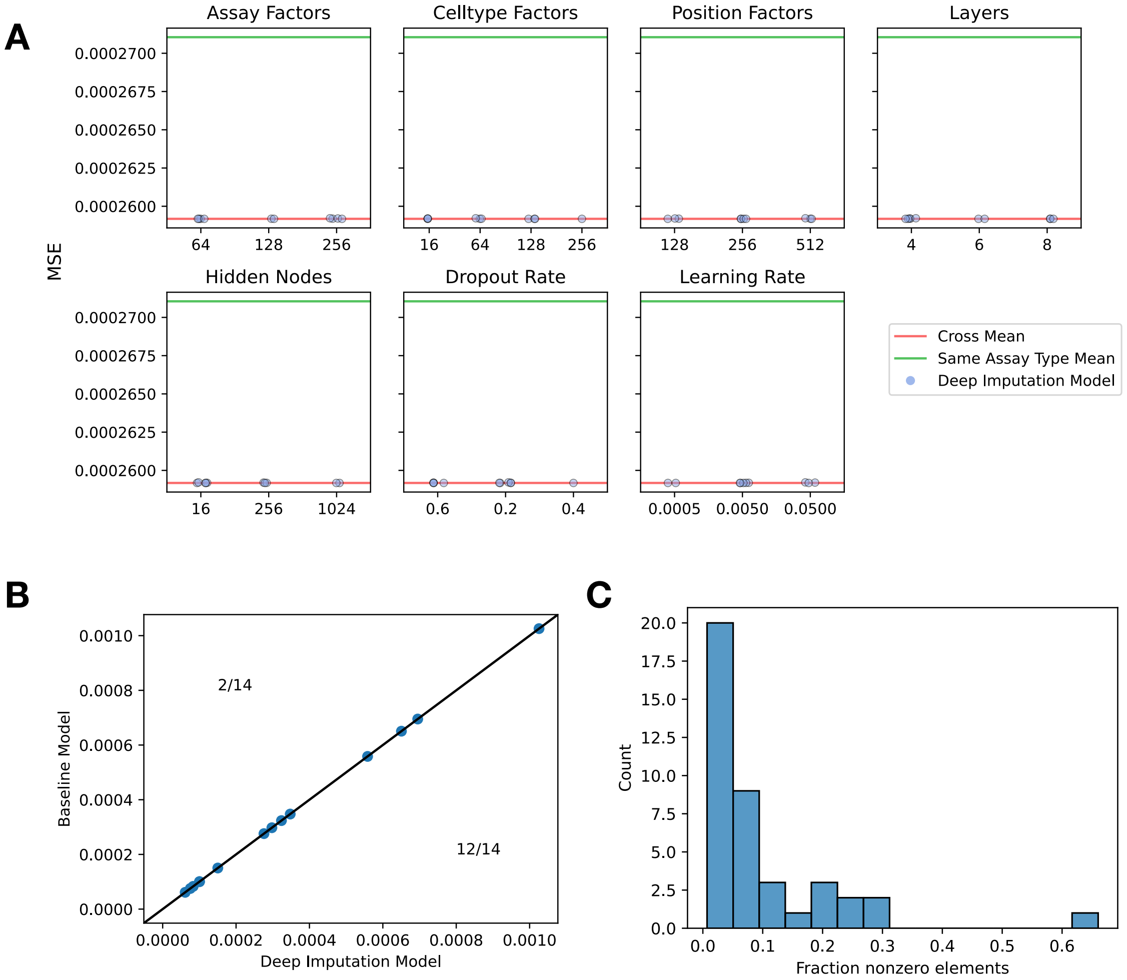

Figure S1: **Sphinx does not offer significant improvement at 10kb resolution** (a) Sphinx was trained using 10 randomly selected settings of seven different hyperparameters. Each panel plots the validation set MSE (x-axis) for various values of a particular hyperparameter (y-axis). MSEs are shown as horizontal lines for the baseline methods: cross-mean (red line) and same assay type (green). Training is done at 10kb resolution on chromosome 19. (b) Test-set MSE is shown comparing the deep imputation model to the cross-mean baseline. The number of points on each side of the $y = x$ line is annotated. (c) A histogram of the fraction on nonzero elements at 10kb resolution for every available dataset is shown.

3.4 Sphinx on larger scale data

To investigate how Sphinx's performance changes when trained from more data, we trained the model on chromosome 1 with the same set-up as when trained on chromosome 19. We kept the sum of the normalized logged counts the be same as in chromosome 19, which meant that on average, the counts when we trained chromosome 1 were lower because there are more bins in chromosome 1. Chromosome 1, after pruning, was 2232×2232, compared to 556×556 in chromosome 19. We reasoned that because there were changes in sparsity and number of bins, we should conduct a hyperparameter search for chromosome 1. Because there were more bins in chromosome 1, training this model was more computationally intensive, and hence we were unable to conduct a full hyperparameter search. We checked around 150 hyperparameter combinations, as opposed to the 500 combinations in chromosome 19.

The deep imputation model outperformed the cross-mean baseline model in validation in 89 hyperparameter combinations, but visually, the most significant improvements were made when the initial learning rate was 0.0005, which was not observed in chromosome 19 (Figure 4A). This difference may be because the average bin had lower counts in chromosome 1 due to our normalization scheme. Unfortunately, we found that in the test set, Sphinx performed similarly or slightly worse than the baseline model using our metrics of the raw MSE, contact decay profile MSE, eigenvector MSE, and insulation score MSE (Figure 4B). We confirmed visually that the model seemed to have converged (Figure 4C). We hypothesize that the decreased performance compared to chromosome 19 may be due to an increase in sparsity in chromosome 1 (Figure 4D), or because the hyperparameter search was not as deep.

We further attempted to run Sphinx at 10kb on chromosome 19 (Section S1); however, we suspect that many of the same issues we experienced with chromosome 1 were exacerbated at 10kb resolution. Namely, there was increased sparsity at 10kb resolution and due to computational constraints, we did not conduct as extensive of a hyperparameter search.

We also added a subsection describing side-by-side examples of where Sphinx did better than the mean baseline and an alternative, convolutional architecture, which is reproduced in our response to Reviewer 3.

5. Once high resolution maps are imputed, it would be great to assess the accuracy of predicted loops or strides.

In response to this suggestion, we used the HiCDetectLoops package [**?**] to detect loops from observed, mean model, and Sphinx predictions at 10kb resolution. We transformed our contact matrices back into raw counts by transforming $\exp(y) - 1$ for both the observed and predicted matrices. We used the following command, which uses the recommended settings from the HiCDetectLoops documentation, including the following parameters: `--maxLoopDistance 2000000 --windowSize 10 --peakWidth 6 --pValuePreselection 0.05 --pValue 0.05`. We detected loops in the observed and predicted matrices for chromosome 19 for each of the 14 entries in our test set.

When using the HiCDetectLoops recommended settings on the WTC-11/ChIA-PET RNA Pol II data, we detected 2 loops in the observed matrices, 9 loops in the Sphinx imputations, and 7 in the mean model. We considered loops to be the same if the start and end of the loops were within 50kb of each other. None of the loops were shared between the observed and either the mean or the Sphinx predictions (Table 1); however, one loop was detected within 1 Mb in both the observed and the imputed matrices at 40Mb. All loops detected in the mean model were also detected in the Sphinx prediction. We also detected 18 loops in the observed WTC-11/ChIA-PET CTCF experiments and 4 loops in the HeLa/DNAse experiments, but no loops were detected in the imputed matrices. We detected 13 loops in the imputed mean model and Sphinx GM12878/DNA SPRITE matrices, but no loops were detected in the observed data. The same contact matrices had loops detected using either the recommended or relaxed HiCDetectLoops commands.

We also used relaxed settings in HiCDetectLoops to attempt to find additional loops, changing maxLoopDistances from 2,000,000 to 5,000,000, and the p-value parameters from 0.05 to 0.1. However, using this approach we still found no matching loops between the observed and imputed matrices using either Sphinx or the mean model.

The discussion section is a bit thin too. For instance, the authors need to explain better when their method predict accurately and when it does not, and the different factors explaining bad

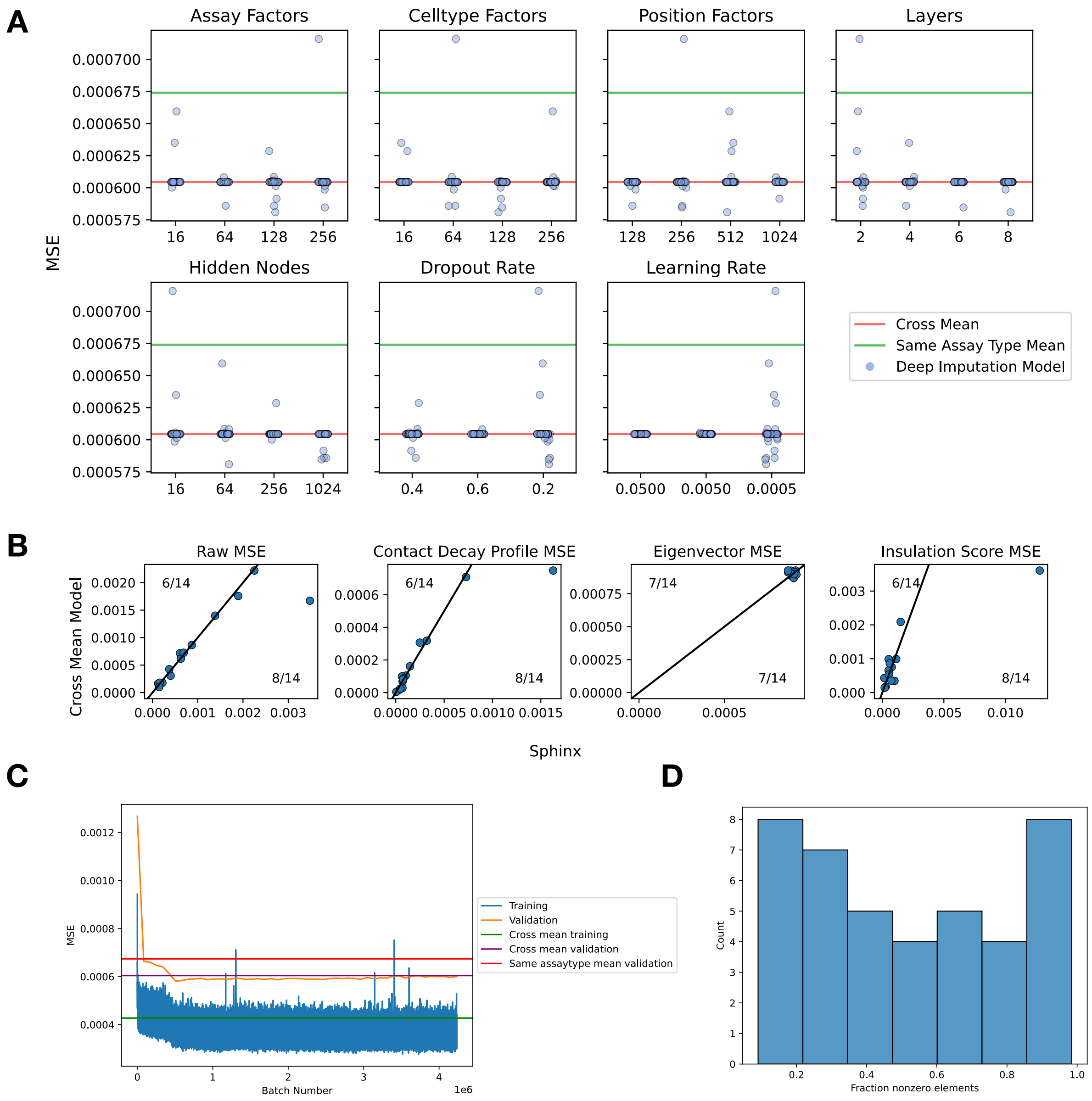

Figure 4: **Sphinx outperforms the baseline in validation data, but not in test data.** (A) We conducted a hyperparameter search using the standard Sphinx model for chromosome 1 with around 150 hyperparameters tested. (B) Raw MSE, contact decay profile MSE, eigenvector MSE, and insulation score MSE are compared between Sphinx and the cross-mean model, similarly to in Figure **??**. (C) The training curve, validation curve, and baselines for the best performing model on chromosome 1. The training curve is a rolling average of 10 batches. (D) A histogram of the fraction of nonzero elements in all data samples in chromosome 1.

Assay	Biosample	Observed loops	Sphinx Predicted loops	Sphinx Intersection	Mean model Predicted loops	Mean model Intersection
in situ ChIA-PET RNA Pol II	WTC 11	2	9	0	7	0
in situ ChIA-PET CTCF	WTC 11	18	0	0	0	0
HeLA	DNAse	4	0	0	0	0
DNA SPRITE	GM12878	0	13	0	13	0

Table 1: **Mean model and Sphinx loop predictions do not agree with the observed loops predictions.** We report all test-set experiments that showed any loop calls, as well as the number of loops that were in the intersection between Sphinx and observed or the number of loops in the intersection of the mean model and observed data, where loops were considered the same if their starts and ends were both within 50kb of each other. All other samples had no detected loops.

predictions in some situations.

The discussion section has been augmented to explain some of the cases in which the method might predict accurately or not:

Each distinct 3D contact map assay provides unique insights into the biology of the cell type that it is used on. Some methods, such as in-situ Hi-C, micro-C, or DNase Hi-C, aim to provide global contact maps. Other methods, such as ChIA-PET or PLAC-seq, focus on interactions mediated by particular proteins. DNA SPRITE includes contacts that are physically more distant, such as in the case of nuclear bodies. These distinct purposes can provide complementary insights.

Although many chromatin 3D contact maps have been collected for combinations of assays and human cell types, there are still many combinations that have not been collected. This missingness motivates a computational method to impute unobserved experiments in order to generate hypotheses and enable downstream analyses that require complete observations. We first developed baseline methods that take the average of experiments either using only the same assay, the same cell type, or either. We then improved on these baseline methods using our machine learning model to predict areas where the baseline method was most likely to be incorrect. We demonstrated that we improved upon the baseline methods in many of the validation cases and more often than not in the test set (Figures **??**A,B).

In our matrix of 88 potential contact maps from the 4DN web portal, we produced imputed experiments for the 47 unobserved experiments (Figure **??**). We then conducted an analysis to determine similarities between assays and cell types across contact decay profile, eigenvector, and insulation score (Figure **??**). These analyses identify characteristics that are unique to individual cell types and assays. We demonstrated that without the imputed data, some comparisons cannot be made due to insufficient data. Furthermore, we demonstrated that some aberrant correlations that were found in the unimputed data were tempered in the imputed data.

A limitation of this work is that the number of available experiments is currently somewhat small, limiting both the training and evaluation of such models. We found that increasing the number of training experiments was associated with a decrease in the MSE (Figure **??**). However, we anticipate that as the cost of sequencing continues to decrease the number of available experiments, and their quality, will increase over time. In parallel, we expect that the number of biosamples of interest will continue to increase as methods for isolating cells from conditions of interest become more sophisticated. As such, we anticipate that methods such as Sphinx will become increasingly valuable.

We also added a section showing some side-by-side examples of the Sphinx and mean model predictions, as requested by Reviewer 3.

The github repo lacks a fully explained readme. For the moment, nothing is done for a user

to properly use the tool. Also a notebook would be appreciated.

The github page has been improved. The source code has been documented more extensively than previously, and a tutorial showing an example of how to use Sphinx has been added. It is available at `https://github.com/Noble-Lab/Sphinx`.

Minor revisions

Fig3 and 4 can be easily merged into one fig. There is so much empty space that be reduced by reducing the plot size and increasing the letter size.

Thank you for this suggestion. The figure has been merged into one Figure 3.

"Our machine learning model to predict areas where the baseline method was most likely to err", err?

We have updated the wording here to say "We then improved on these baseline methods using our machine learning model to predict areas where the baseline method was most likely to be incorrect."

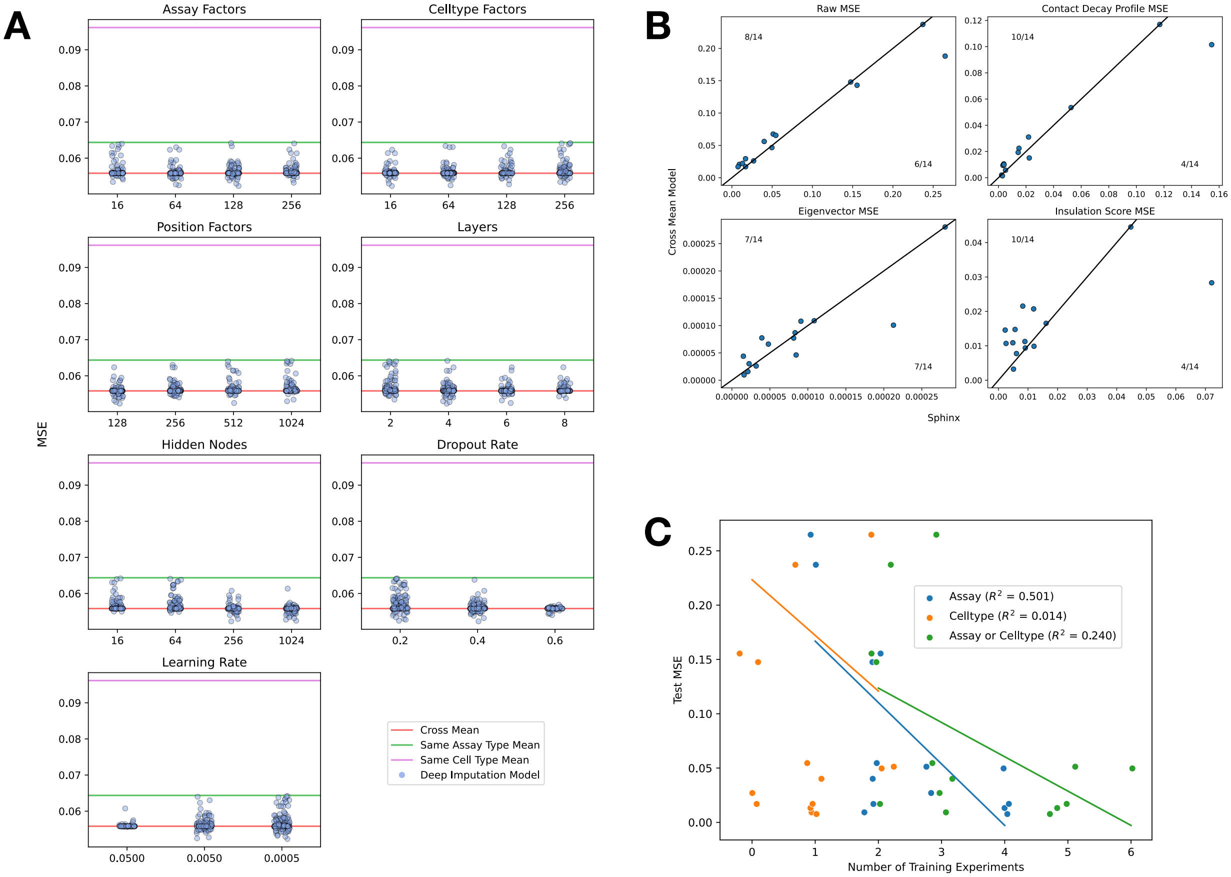

Figure 3: **Sphinx outperforms mean model baseline**. (a) Sphinx was trained using 500 randomly selected settings of seven different hyperparameters. Each panel plots the validation set MSE (x-axis) for various values of a particular hyperparameter (y-axis). MSEs are shown as horizontal lines for the three baseline methods: cross-mean (red line), same cell type (magenta), and same assay type (green). All analyses are based on data from chromosome 19. (b) Each panel plots the test set MSE achieved by Sphinx (x-axis) versus the cross-mean baseline (y-axis). Each point corresponds to one of the 14 assay/cell type combinations in the test set. (c) A comparison of the number of training experiments that shared assay (blue), cell type (orange), or either assay or cell type (green) versus the MSE of the test set example. Each point is one test set example. The x-axis is jittered for visibility. Least squares lines are shown along with their associated R^2 values.

Reviewer 2

The work proposed a new method named Sphinx, a deep tensor factorization method to impute unseen contact maps given only a collection of available contact maps. Sphinx is developed based on the method Avocado, which is used to impute 1D epigenomic data. However, some key details are missing and there is no benchmark with existing methods for a similar purpose. I have some comments for helping improve the quality of the work.

1. One big limitation as the author mentioned is computational constraints. Can the author provide more details regarding the constraints? One related question is "We only trained the model on one chromosome at 100 kb resolution due to computational constraints. Chromosome 19 was chosen due to its moderate size." Would the results vary for other chromosomes? That is, the results presented on chr19 may be biased towards the selection of one particular chromosome?

We managed to run the model at 10kb resolution (described in Reviewer 1, Comment 1), and also ran the model on chromosome 1. We found that we had improved validation MSE over the baseline model in chromosome 1, but on the test set, there was similar performance to the baseline model. We described these findings in a new results section, which is reproduced in our response to Reviewer 1.

Moreover, some hic technology may not have 100kb resolution and some have higher resolution, how the author deal with this to make all resolution 100kb?

All of the datasets that we used were available via the 4DN web portal at 100kb resolution. If a given dataset were available only at finer resolution, then it would be straightforward to aggregate reads to achieve a coarser resolution. On the other hand, if a given assay produces data that is coarser than 100kb resolution, our method would not be able to make use of such data.

2. How do the data processing or the method address batch effect across biosamples (technical or biological replicates) and resolution difference from different HiC technologies when performing the imputation?

Because our data does not include any technical or biological replicates, we are unable to include a method to address batch effects. We attempt to minimize differences by using a normalization procedure to make the total number of log counts in each experiment to be the same (Section **??**).

3. Compared to 1D epigenomic data, as used by Avocado, the limitation for imputing HiC data, is the tissues/cell types and overall sample size is far fewer compared to 1D epigenomic data. Therefore, the tensor-based imputation method may be sensitive to the sample size. Would the author evaluate the performance of the proposed methods based on different sample sizes?

Thank you for this suggestion. We conducted this experiment and found that as we lower the number of training samples, the validation and test MSE increase. We have included this figure in the Supplement. The figure is shown here as Figure S2. We added the following text to Section **??**.

We also confirmed that removing training examples increased the validation MSE (Figure S2)

4. The proposed work should be compared with existing methods as some of those mentioned in the introduction. Other tissue/cell type-specific training HIC model can used for cross-tissue/cell type prediction in a transfer learning way. Only with compared to other methods in such a way, Sphinx can be showed is effective in 3D map imputation.

To our knowledge there is no way to compare to the other methods, since they do not take into account other assays and cell types as Sphinx does. For example, Akita, DeepC, and Orca take sequence as input, whereas Sphinx does not. Epiphany uses 1D epigenomic tracks that we do not have access to. Likewise, for DeepChIA-PET, ChIP-seq data is required. We elaborated on this point by adding the following text in Section **??**.

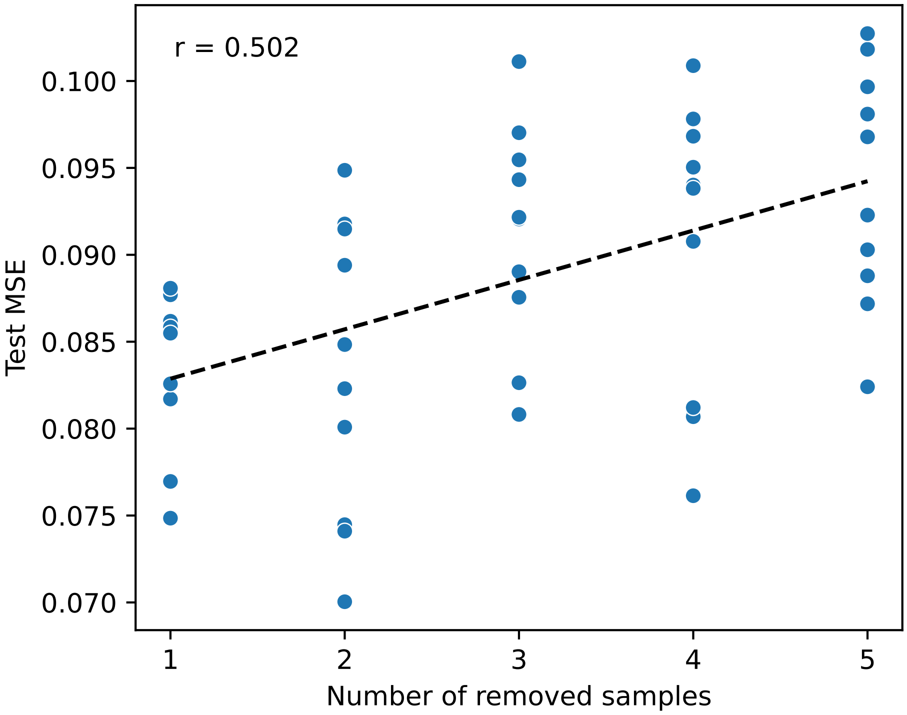

Figure S2: **MSE increases as the number of training examples decreases**. One to five samples were randomly removed (X-axis) from the training data so that there was always at least one training example of each celltype and assay were still present in the training set, and the and test MSE was recorded. This was done for 10 random starting seeds.

Due to the key differences between Sphinx and existing methods described in Section **??**, there are no appropriate alternate methods to compare with Sphinx.

5. There is no way to reproduce the results and run the software as provided in the github.

The github page has been improved. The source code has been documented more extensively than previously, and a tutorial showing an example of how to use Sphinx has been added. It is available at `https://github.com/Noble-Lab/Sphinx`.

Reviewer 3

This manuscript introduces Sphinx, an MLP that imputes missing Hi-C profiles from 4DN experiment grid. However, it seems to perform similarly to the average baseline. This leads to the question of why using a model if we can just calculate the average. The author needs to better address the motivation of this study.

1. The author compared the Sphinx imputation result with the cross-average baseline and there doesn't seem to be noticeable improvements from the average baseline (Figure 4). This leads to two questions: 1. Is the average profile already good enough for most of the downstream Hi-C analysis? For example, cell-type-specific TAD/loop calling. If it is, why do we need a model?

Although the improvements are marginal, we believe that the framework for Sphinx allows for further improvements as we observe more data. We noted that as we increase the number of samples used for training, we decrease the MSE in the Sphinx model. This may not be the case for the mean model. Furthermore, Sphinx provides latent celltype, assay, and position factors that have potential to be used for downstream analyses.

2. If the average profile is not good enough, can the prediction result from Sphinx enable these tasks? Showing a few side-by-side examples where Sphinx did better than baseline would be good to motivate the study.

We added some side-by-side examples in Section 3.4 provide some insight into specific examples where Sphinx did better than the baseline model in the test data and can serve as motivation.

3.3 Imputation reveals hypotheses for contact decay profiles and insulation score

Using our held out validation and test datasets, we can verify that hypotheses generated by our imputations are plausible. For example, when we consider contact decay profiles (Figure ??), we observe that for micro-C in H1-hESC, which is a test set example, the Sphinx imputed contacts near the diagonal are less prominent than the mean-model imputed contacts near the diagonal. Previous work has found that embryonic stem cells tend to exhibit a greater number of long-distance contacts than more differentiated cell types [1]. In our test example, Sphinx accurately predicts this phenomenon. Sphinx also accurately imputes less frequent short-range contacts for micro-C in the WTC-11 cell line, which is an induced pluripotent stem cell line. These two observations suggest that Sphinx may be able to infer characteristics of less differentiated cell lines, which may otherwise be drowned out in the cross-mean average baseline method. Sphinx also predicts, for the unobserved micro-C experiment in GM12878, increased short-range interactions compared to the cross-mean baseline, which may be reflective of the fact that GM12878 is a more differentiated lymphoblast cell line, compared to the embryonic stem cells in either H1-hESC or WTC-11. Interestingly, Sphinx predicts that the unobserved micro-C experiment for IMR-90, which is a fibroblast cell line, has decreased close-range contacts compared to the baseline.

Another example can be seen in the insulation scores for dilution Hi-C (Figure ??). For one of the test set examples, GM12878, we observe several regions where Sphinx accurately predicts lower insulation scores than the mean model. Although in the CC-2551 example, Sphinx predicts a very similar profile to the mean model, these two examples suggest that insulation scores computed from Sphinx results are plausible. We observe that there are various regions in the H1-hESC cell type where Sphinx predicts lower insulation scores than the mean model, which may be an area that warrants further investigation.

2. The resolution of 100kb is capturing mainly compartments, and very large TADs. The more interesting structure for example loops are usually at finer resolution. Can this method

be applied at 10kb or 1kb resolution to enable realistic applications?

Please see response to Reviewer 1, Comment 1.

3. The author used a MLP which is a very simple architecture. However more recent literatures like Akita, DeepC, Orca, Epiphany and ChromaFold used CNN. It would be good to see if CNN achieves better performance.

Thank you for this suggestion. We implemented a convolutional architecture per this suggestion and found that it did not improve upon the performance of the original Sphinx model. We added a new subsection of the results describing this experiment.

3.5 Experimenting with a convolutional architecture

A convolutional architecture (Figure 11A) was also implemented as an alternative to the fully connected layer. This architecture is similar to the main Sphinx model except that that neighboring positions were convolved with each other before being concatenated with the cell type and assay factors. If the position of interest was too close to the end of the chromosome, then we set the windowed positions outside of the chromosome to be 0. We trained the convolutional model using chromosome 19 at 100kb resolution using the best-performing hyperparameters for the base Sphinx model (16 celltype factors, 128 assay factors, 128 position factors, 128 distance factors, 256 hidden nodes, 4 hidden layers, with a dropout of 0.4, and an initial learning rate of 0.0001). We set a window of 10 positions before the central position and 10 positions after, for a total of 21 sets of position factors being entered into the convolutional neural network. We used two 2D convolutional layers. The first had one input channel, three output channels, and a kernel size of 21. The second had three input channels, one output channel, and a kernel size of one. The output of these two convolutional layers was input into the fully connected layers, and the rest of the model was the same as in the main Sphinx model. The model was trained for 50 epochs, and the iteration with the best validation error was kept.

We used the Sphinx model to make predictions on each of the test set examples, as in Figure **??**, and we compared to the MSE from the mean model and the convolutional Sphinx model. We found that in terms of raw test-set MSE and contact decay profile MSE, the convolutional Sphinx model performed marginally worse than the mean model. The eigenvector MSE was better in the Sphinx model for all cases, and the insulation score was worse in most cases (Figure 11B). We determined that the average Sphinx validation MSE was overall improved upon the average mean model validation MSE and that the model appears to have converged upon visual inspection (Figure 11C). We noted that these results do not represent large improvements over the standard Sphinx model and hence did not continue to pursue the convolutional model.

4. Chromosome 19 is one of the smallest chromosomes. It would be great if the authors could show if the model can impute at larger scale chromosomes for example chromosome 1.

Please see response to Reviewer 2, Comment 1.

Other small edits

We realized we did not mention the distance factors that were part of the model architecture and included a line describing this in the text, and updated the figure accordingly (Figure 2).

We further include a set of distance factors encoding the distance between the two genomic positions

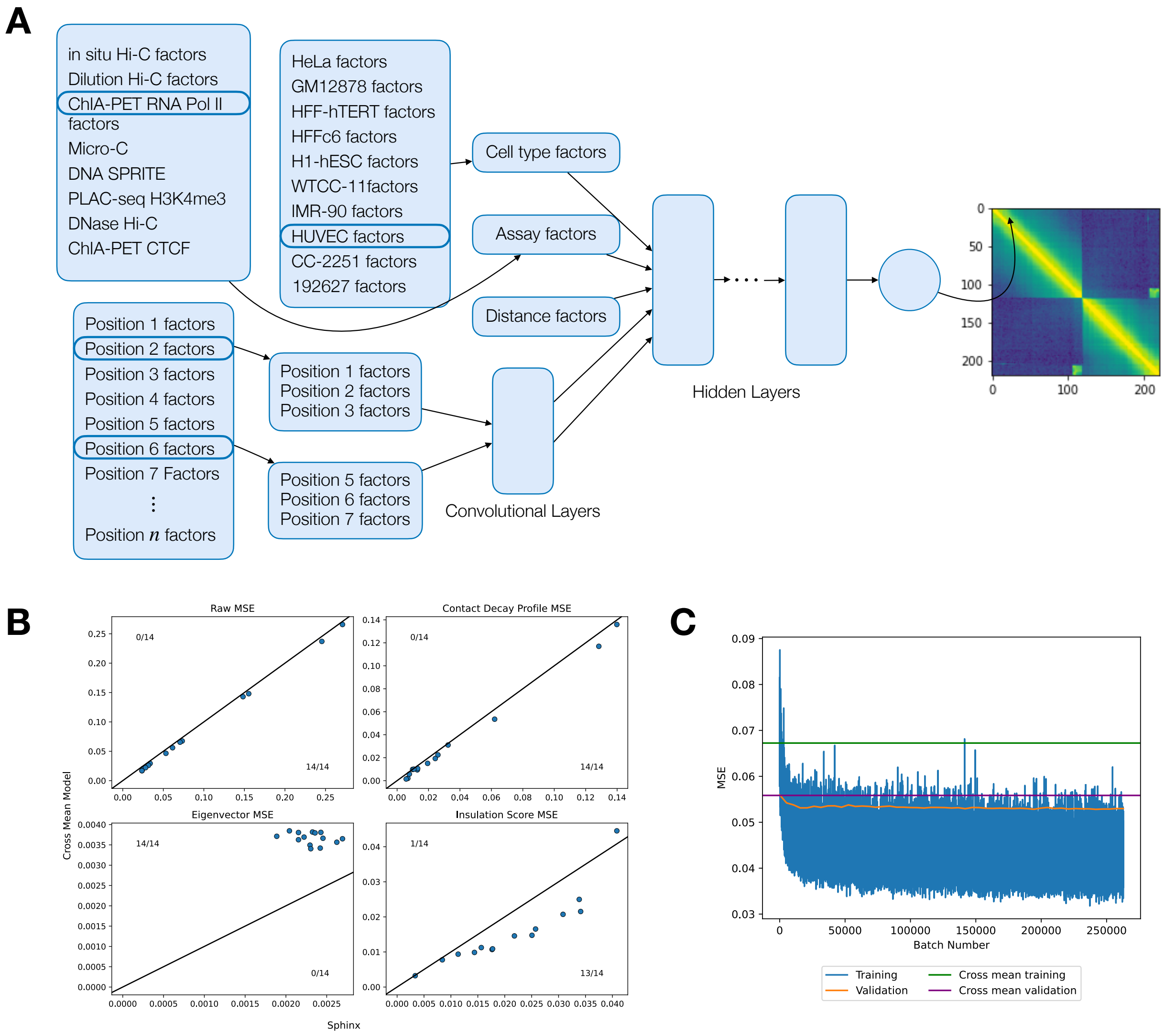

Figure 11: **Convolutional Sphinx architecture does not improve performance over standard architecture** (a) Cell type and assay factors are input into the hidden layers as in Figure 2. Position factors are first convoluted in a window of neighboring factors. The window size is shown as 1 in the figure as an example. The result of the convolution is input into the hidden layers. (b) A comparison of the raw, contact decay profile, eigenvector, and insulation score MSEs. The black line indicates $y = x$. (c) The training curve (rolling average over 10 batches) and validation curve (plotted per epoch) are shown and compared to baselines.

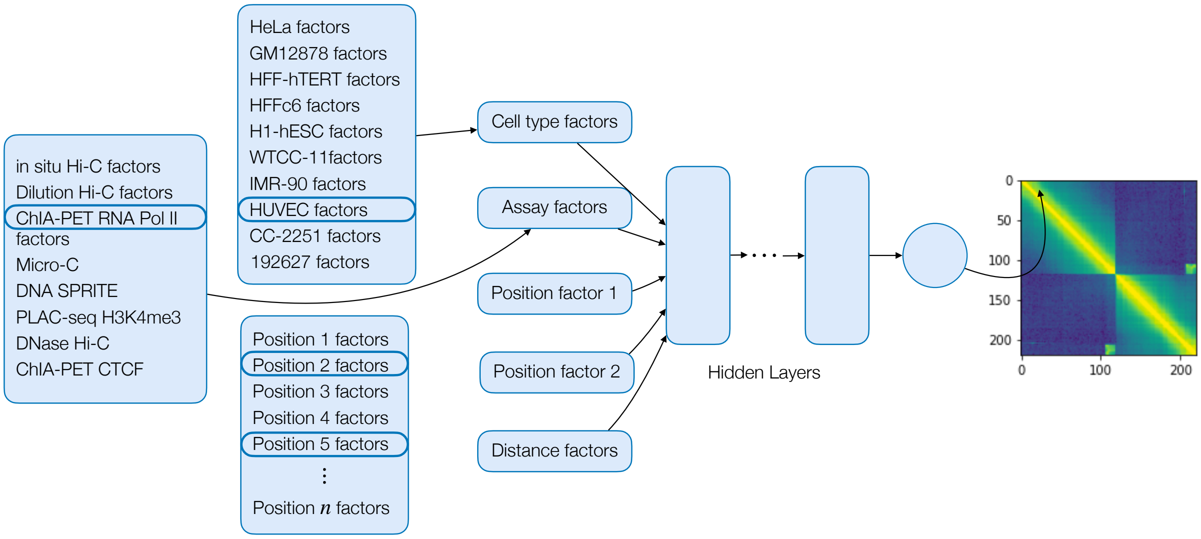

Figure 2: **The Sphinx model.** Each dimension of the 4D input is encoded using latent factors, and the concatenated factors are processed by a multi-layer perceptron. The final output of the model is the predicted normalized contact count.

References

[1] Boyan Bonev, Netta Mendelson Cohen, Quentin Szabo, Lauriane Fritsch, Giorgio L Papadopoulos, Yaniv Lubling, Xiaole Xu, Xiaodan Lv, Jean-Philippe Hugnot, Amos Tanay, et al. Multiscale 3D genome rewiring during mouse neural development. *Cell*, 171(3):557–572, 2017.
