# Supplementary material for: Predicting chromatin conformation contact maps
Source: PLoS One. 2025 Sep 29;20(9):e0331124. doi: 10.1371/journal.pone.0331124 (PMC12478923; doi:10.1371/journal.pone.0331124)
Supplement: Attachment — Submitted filename: plos-one-response2.pdf [file pone.0331124.s003.pdf]

We appreciate the constructive suggestions made by the reviewers. Please see below for our detailed responses to each of the critiques. Where applicable, we have included the revised text in blue.

Reviewer 1

In this work, the authors introduce Sphinx, a model designed to impute chromatin conformation contact maps. Sphinx framework predicts missing maps based on a collection of existing datasets from the 4D Nucleome (4DN) project. The authors evaluate Sphinx using cross-validation and compare it to baseline averaging methods. The analyses focus on contact decay profiles, compartments (eigenvectors), and topologically associating domains (TADs). I will try to not be repetitive given the comments of the other 3 reviewers and the authors' responses. I have some comments and questions that could help to clarify some aspects of the text and the results:

Thank you for your helpful comments, which we have addressed below.

I suggest the authors to expand the discussion on the limitations of the tool to impute loops. It may require a new layer to be included in the network. It is not necessary to do it at this point but it would be beneficial to point this out.

We have added a supplementary section regarding loop calls, and it is reproduced below.

1 Imputing Loops

Another important aspect of HiC data is predicting loops, which are regions of the genome that are brought together in three dimensional space through loop extrusion. These loops have biological significance in modulating gene expression, and many methods have been proposed to detect loops [1, 2, 3]. It is hence of interest to determine whether loops can be imputed through Sphinx. We used the HiCDetectLoops package [1] to detect loops from observed, mean model, and Sphinx predictions at 10kb resolution. We transformed our contact matrices back into raw counts by transforming $\exp(y) - 1$ for both the observed and predicted matrices. We used the following command, which uses the recommended settings from the HiCDetectLoops documentation, including the following parameters: `--maxLoopDistance 2000000 --windowSize 10 --peakWidth 6 --pValuePreselection 0.05 --pValue 0.05`. We detected loops in the observed and predicted matrices for chromosome 19 for each of the 14 entries in our test set.

When using the HiCDetectLoops recommended settings on the WTC-11/ChIA-PET RNA Pol II data, we detected 2 loops in the observed matrices, 9 loops in the Sphinx imputations, and 7 in the mean model. We considered loops to be the same if the start and end of the loops were within 50kb of each other. None of the loops were shared between the observed and either the mean or the Sphinx predictions (Table 1); however, one loop was detected within 1 Mb in both the observed and the imputed matrices at 40Mb. All loops detected in the mean model were also detected in the Sphinx prediction. We also detected 18 loops in the observed WTC-11/ChIA-PET CTCF experiments and 4 loops in the HeLa/DNAse experiments, but no loops were detected in the imputed matrices. We detected 13 loops in the imputed mean model and Sphinx GM12878/DNA SPRITE matrices, but no loops were detected in the observed data. The same contact matrices had loops detected using either the recommended or relaxed HiCDetectLoops commands.

We also used relaxed settings in HiCDetectLoops to attempt to find additional loops, changing maxLoopDistances from 2,000,000 to 5,000,000, and the p-value parameters from 0.05 to 0.1. However, using this approach we still found no matching loops between the observed and imputed matrices using either Sphinx or the mean model. Because loops are a high resolution feature, we expect that further refinement may produce more concordant loop calls.

I would recommend a quick additional analysis to benchmark when comparing the maps. Could the authors calculate the Pearson as a function of genomic distance (for example `10.`

Assay	Biosample	Observed loops	Sphinx<br>Predicted loops	Sphinx<br>Intersection	Mean model<br>Predicted loops	Mean model<br>Intersection
in situ ChIA-PET RNA Pol II	WTC 11	2	9	0	7	0
in situ ChIA-PET CTCF	WTC 11	18	0	0	0	0
HeLA	DNAse	4	0	0	0	0
DNA SPRITE	GM12878	0	13	0	13	0

Table 1: **Mean model and Sphinx loop predictions do not agree with the observed loops predictions.** We report all test-set experiments that showed any loop calls, as well as the number of loops that were in the intersection between Sphinx and observed or the number of loops in the intersection of the mean model and observed data, where loops were considered the same if their starts and ends were both within 50kb of each other. All other samples had no detected loops.

`1073/pnas.161360711` - SI and `10.1093/nar/gkaa818` - fig.4 G)?

We added this analysis in the supplement and it is reproduced below. We also discuss this in Section 3.1.

Did the authors try to impute inter-chromosomal maps? That would be interesting to comment on that result or comment as a potential improvement of the model in later versions.

We added a comment in the discussion reproduced below.

As data becomes more robust, we envision that it may become possible to impute interchromosomal contacts, which we did not attempt with Sphinx.

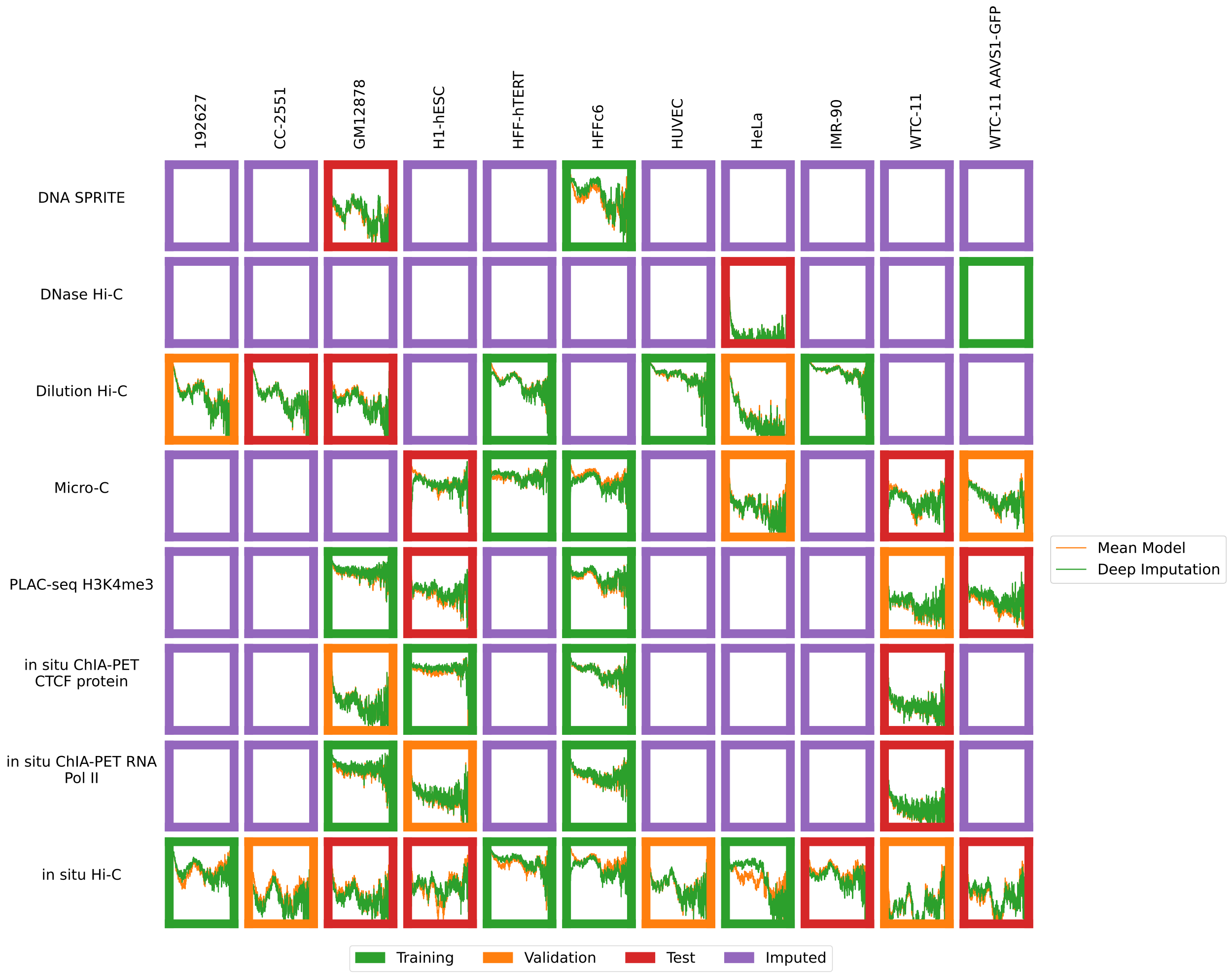

Figure 1: **Pearson correlation by distance.** Pearson correlation (y-axis, ranging from 0-1) is plotted against the genomic distance of the two positions (x-axis) [4]. The correlation is between either the mean model (orange lines) or Sphinx (green lines) compared to the observed contact matrices. The border of each panel indicates the data split that the matrix was assigned to.

2 Reviewer 2

In the present manuscript by Min et al., the authors introduce Sphinx, a computational method that uses machine learning to impute missing chromatin capture contact matrices using existing experimental data as input. The main data source for the development and testing of Sphinx are datasets from the 4DN Nucleome. Existing methods use either just DNA sequence as input or require at least some additional experimental data of epigenomic profiling to exist. In contrast, Sphinx imputes contacts by using as sole input a collection of contact maps. The authors present the data, testing methodology and their conclusions appropriately. Importantly they discuss the limitations of the tool in its current state and the amount of data available.

The available submitted PDF by the authors already contains the responses to three reviewers and manuscript modifications. I understand that this correspond to a previous review round for publication at PLoS One and that these responses should be considered, but if so this was not apparent to me from the invite or the instructions provided.

Sorry that this was not made clear. Our manuscript was previously reviewed at *PLOS Computational Biology* and then transferred (with reviews) to *PLOS One*.

The authors have addressed most of the reviewers concerns and extensibly modified the manuscript. My reviewer recommendation is a minor revision with my specific comments below:

Figure 1. The introduction states "among the 88 possible contact maps, 4DN has carried out 41. Our goal is to accurately impute the missing 47 contact maps". However, the figure legend reads "44 non-missing contact maps".

Thank you for pointing this out. The caption was incorrect and this has been updated.

Figure 2. Scheme is unclear, text labels are inconsistently positioned, some boxes are empty with unclear meaning, why positions are highlighted with blue circles is not described

Thank you for catching this. The figure has been updated as well as the caption and this is reproduced below.

Page 6. MSE (mean squared error) is an undefined acronym, even though very common I think the authors should define it once when first mentioned in the text.

This has been fixed.

Figure 4 is referenced in the text out of order (after Figure 9), figure numbers should be arranged in the order they are referenced in the text

This has also been fixed.

There are some missing references in reviewer responses (both to papers, sections and figures in the manuscript), probably errors in the LaTeX formatting

We have fixed the missing references.

References

[1] Joachim Wolff, Rolf Backofen, and Björn Grüning. Loop detection using hi-c data with hicexplorer. *Gigascience*, 11:giac061, 2022.

[2] Abbas Roayaei Ardakany, Halil Tuvan Gezer, Stefano Lonardi, and Ferhat Ay. Mustache: multi-scale

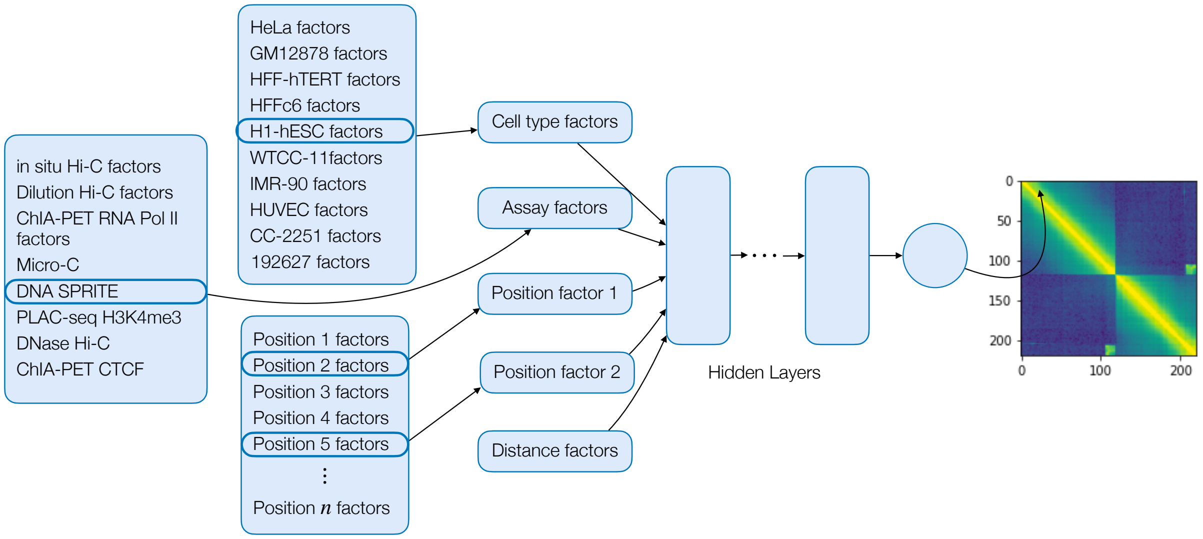

Figure 2: **The Sphinx model.** Each dimension of the 4D input is encoded using latent factors, and the concatenated factors are processed by a multi-layer perceptron. The figure shows an example of predicting the contact between positions 2 and 5 in DNA SPRITE H1-hESC. The final output of the model is the predicted normalized contact count.

detection of chromatin loops from Hi-C and micro-C maps using scale-space representation. *Genome Biology*, 21:1–17, 2020.

[3] N. C. Durand, M. S. Shamim, I. Machol, S. S. Rao, M. H. Huntley, E. S. Lander, and E. L. Aiden. Juicer provides a one-click system for analyzing loop-resolution Hi-C experiments. *Cell Systems*, 3(1):95–98, 2016.

[4] Vinícius G Contessoto, Ryan R Cheng, Arya Hajitaheri, Esteban Dodero-Rojas, Matheus F Mello, Erez Lieberman-Aiden, Peter G Wolynes, Michele Di Pierro, and José N Onuchic. The nucleome data bank: web-based resources to simulate and analyze the three-dimensional genome. *Nucleic Acids Research*, 49(D1):D172–D182, 2021.
